# Toward an Assessment of the Global Inventory of Present-Day Mercury Releases to Freshwater Environments

**DOI:** 10.3390/ijerph14020138

**Published:** 2017-02-01

**Authors:** David Kocman, Simon J. Wilson, Helen M. Amos, Kevin H. Telmer, Frits Steenhuisen, Elsie M. Sunderland, Robert P. Mason, Peter Outridge, Milena Horvat

**Affiliations:** 1 Department of Environmental Sciences, Jožef Stefan Institute, Ljubljana 1000, Slovenia; milena.horvat@ijs.si; 2 Arctic Monitoring and Assessment Programme (AMAP) Secretariat, Oslo N-0349, Norway; s.wilson@inter.nl.net; 3 Department of Environmental Health, Harvard School of Public Health, Boston, MA 02215, USA; helen.amos@gmail.com (H.M.A.); ems@seas.harvard.edu (E.M.S.); 4 Harvard School of Engineering and Applied Sciences, Cambridge, MA 02138, USA; 5 Artisanal Gold Council, Victoria, BC V8W 1B9, Canada; ktelmer@artisanalgold.org; 6 School of Earth and Ocean Sciences, University of Victoria, Victoria, BC V8P 5C2, Canada; 7 Arctic Centre, University of Groningen, Groningen 9718CW, The Netherlands; f.steenhuisen@rug.nl; 8 Department of Marine Sciences, University of Connecticut, CT 06340, USA; robert.mason@uconn.edu; 9 Geological Survey of Canada, Natural Resources Canada, Ottawa, ON K1A 0E8, Canada; peter.outridge@canada.ca

**Keywords:** mercury, freshwater systems, releases, inventory, global cycling

## Abstract

Aquatic ecosystems are an essential component of the biogeochemical cycle of mercury (Hg), as inorganic Hg can be converted to toxic methylmercury (MeHg) in these environments and reemissions of elemental Hg rival anthropogenic Hg releases on a global scale. Quantification of effluent Hg releases to aquatic systems globally has focused on discharges to the global oceans, rather than contributions to freshwater systems that affect local exposures and risks associated with MeHg. Here we produce a first-estimate of sector-specific, spatially resolved global aquatic Hg discharges to freshwater systems. We compare our release estimates to atmospheric sources that have been quantified elsewhere. By analyzing available quantitative and qualitative information, we estimate that present-day global Hg releases to freshwater environments (rivers and lakes) associated with anthropogenic activities have a lower bound of ~1000 Mg·a^−1^. Artisanal and small-scale gold mining (ASGM) represents the single largest source, followed by disposal of mercury-containing products and domestic waste water, metal production, and releases from industrial installations such as chlor-alkali plants and oil refineries. In addition to these direct anthropogenic inputs, diffuse inputs from land management activities and remobilization of Hg previously accumulated in terrestrial ecosystems are likely comparable in magnitude. Aquatic discharges of Hg are greatly understudied and further constraining associated data gaps is crucial for reducing the uncertainties in the global biogeochemical Hg budget.

## 1. Introduction

Mercury (Hg) is a pollutant of global concern that cycles between the atmosphere, terrestrial and aquatic systems and has been strongly impacted by human activity [1,2]. Understanding Hg inputs to various environmental compartments and their magnitude is an important step in assessing risks for humans and wildlife, and for characterizing the global biogeochemical cycle. Releases of Hg to aquatic systems are especially important, as it is within these environments that toxic methylmercury (MeHg) that bioaccumulates in aquatic food webs is formed [3]. Compared to global atmospheric emission inventories of Hg [4,5,6], direct releases to aquatic systems have been understudied. Horowitz et al. [7] quantified global time-dependent historical releases of commercial Hg to different environmental reservoirs including water but did not develop a resolved spatial inventory of present-day releases to freshwater environments. Other work has focused on the riverine flux of Hg to the oceans [8,9,10] and Hg releases from contaminated systems [11]. Here we present a spatially-distributed, sector-specific inventory of Hg releases to freshwater environments on a global scale to extend understanding of the global distribution of these inputs.

Mercury is released to freshwater systems from a variety of sources and subjected to complex transport pathways [12]. Mercury can enter rivers and lakes directly via atmospheric deposition. Rivers and lakes further receive Hg as a result of releases from industrial sources within their drainage area, as well as indirectly from terrestrial environments via surface runoff, and through soil leaching and erosion. Depending on the soil characteristics, part of this Hg reservoir can be leached to groundwater [13]. Riverine loads are enhanced by river bank erosion as well as remobilization of previously deposited Hg with bed sediments during high water flow, especially in contaminated systems [14,15]. On the other hand, transport of Hg from rivers to coastal environments is diminished by its sedimentation behind various impoundments during transport to the ocean.

Riverine Hg loads from terrestrial systems to the coastal zone as estimated in various studies [8,9,10] vary by a factor of five. These include a higher end estimate of 5500 ± 2700 Mg·a^−1^ by Amos et al. [10], derived using a global gridded inventory based on a review of published Hg measurements taken near river mouths. This estimate is a factor of two or larger than previously estimated values by Cossa et al. [8] (~1000 Mg·a^−1^) and Sunderland and Mason [9] (2040, range 1320 to 2760 Mg·a^−1^), due to the incorporation of recently published data from major Asian rivers. However, recent estimate by Liu et al. [16] suggests lower inputs from rivers in China. On the other hand, terrestrial systems receive Hg through wet and dry deposition that are estimated to be approximately 3000 Mg·a^−1^ [17]. 

**Figure 1:**
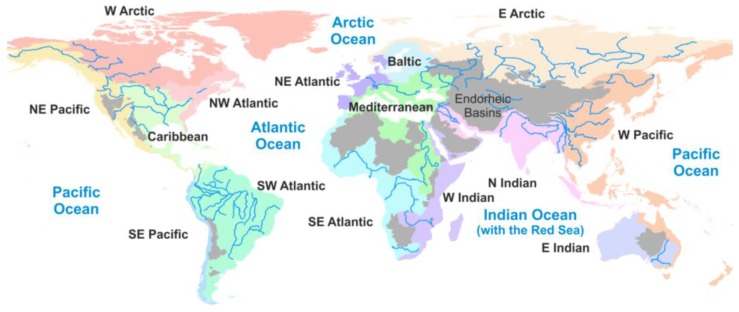
Drainage basins of the principal oceans and seas of the world. Description in blue is used for oceans, and black color is used to denote the 15 drainage basins considered in our inventory. Maps used: World Major Rivers (source: ESRI^®^, Redlands, CA, USA), Drainage Basins (source: compiled by William Rankin (personal communication) based on USGS Hydro1k database (Garretson, SD, USA)).

Our inventory accounts for Hg released from anthropogenic sources, as well as remobilized from natural and anthropogenic Hg previously accumulated in the terrestrial environment. We focus on Hg releases from these sources to adjacent freshwater systems, excluding groundwater due to the lack of quantitative information. In order to avoid double counting, we distinguish between direct Hg releases to freshwaters (i.e., Hg discharged directly into water bodies) and inputs to water bodies from terrestrial environments via runoff, soil erosion and leaching. Our work builds on the inventory prepared by the United National Environment Programme (UNEP) and the Arctic Monitoring and Assessment Program (AMAP) that was part of the UNEP global mercury assessment [18]. The underlying assumptions for estimating Hg releases from diffuse sources are based on general long-term hydro-meteorological conditions, while releases from point sources refer to the 2010 inventory. We qualitatively discuss land management practices such as silviculture and agriculture, and the use and consequent release of Hg in artisanal and small-scale gold mining (ASGM). Quantitative information on Hg releases to surrounding freshwaters from these sources is largely lacking. We compare the relative contributions from individual source categories with those from atmospheric deposition and riverine transport of Hg to oceans. We distribute the inventory geospatially according to the major drainage basins of the world (Figure 1).

## 2. Methods

We distinguish among three types of Hg sources to freshwater environments: (1) primary anthropogenic, (2) soil erosion and runoff not impacted by direct anthropogenic releases (background), and (3) remobilization of Hg from contaminated sites and areas impacted by land management practices. Given the data availability for individual Hg sources, different approaches and assumptions were used to derive releases. Methods used are documented in the following paragraphs, with more details provided in the Supplementary Materials Figure S1, Tables S1 and S2. To the extent possible we compare our results with independent release estimates and recognize that there is substantial uncertainty in some of these initial estimates. Inventory results are summarized for 15 drainage basins of the principal oceans and seas of the world (Figure 1). We compare these numbers with estimates of more general Hg inputs to ocean margins via rivers and atmospheric deposition in order to provide context for the discussion. While we acknowledge that the fate of aquatic Hg depends greatly on its chemical form and partitioning between dissolved and particulate phases, insufficient data are available to quantify speciated releases, such as the inputs of MeHg.

### 2.1. Primary Anthropogenic Release Estimates

Primary anthropogenic Hg sources consist of Hg released with the effluents leaving production sites where Hg is intentionally or unintentionally used and/or present in products and processes. For sectors covered by the UNEP Toolkit [19,20] (chlor-alkali industry, oil refining, large scale Au and non-ferrous metal production, disposal of Hg-containing products) the UNEP Toolkit provides “distribution factors” that proportionally “distribute” total Hg releases between emissions to air and releases to water and land. We used these factors together with the most recent Global Mercury Assessment (GMA) atmospheric Hg emission inventory [18] to calculate the corresponding magnitudes of releases to water (see Supplementary Material Table S1 for details). Similarly, the methods used to geospatially distribute air emissions [21] were then applied to the aquatic release estimates to achieve the geospatial-distribution of the aquatic releases. The combined approach is described further in the Figure S1.

In addition to industrial effluents, we consider Hg releases associated with domestic water use, a sector recently recognized as important also by Liu et al. [22]. We derive our wastewater estimates from reported Hg concentrations in wastewater effluents and global distribution of domestic water use using gridded dataset developed for the Millennium Ecosystem Assessment Series [23]. For each basin we select a ratio of treated to untreated wastewater reaching water bodies based on information presented by UNEP [24] and Sato et al. [25]; 90:10 for Baltic and NE Atlantic, 80:20 for NW Atlantic, E Indian and SW Pacific (part draining Australia and New Zealand), 70:30 for NE Pacific, Caribbean (part draining North America) and W Arctic, 60:40 for Mediterranean, 50:50 for E Arctic and Endorheic regions, 30:70 for N Indian, SE Pacific, SW Atlantic, W Pacific and Caribbean (part draining Central America), 20:80 for SE Atlantic and W Indian. We use a wastewater Hg concentration in 100–500 ng L^−1^ range for untreated waste-water and a Hg concentration range of 5–20 ng L^−1^ for water treated in waste-water treatment plants (WWTPs) [26,27,28].

We discuss releases from ASGM based on the global inventory prepared by AMAP/UNEP [18], which is based on the improved methodology of Telmer and Veiga [29] and the continuous updates delivered to a publically available (http://mercurywatch.org) database. The methodology combines understanding of ASGM activities, especially knowledge about practices used in individual countries, with field evidence and variety of secondary information sources [18]. Exact locations where ASGM activities are taking place are not always known. Moreover, as these sites are distributed globally in zones with highly variable hydro-meteorological conditions, it is reasonable to expect that inputs to local aquatic systems will significantly differ from site to site. In the absence of detailed information, we use a semi-quantitative approach to estimate potential release of Hg to aquatic systems for individual country based on their susceptibility to erosion. We classify countries into three groups based on the amount of surface runoff [30]: countries with a very dry climate (<100 mm·a^−1^) where remobilization of Hg to aquatic systems can be considered negligible, countries with a very humid climate (>1000 mm·a^−1^) where such inputs can be important, and others that fall in-between these two classes.

### 2.2. Background Releases from Terrestrial Systems

Background releases are comprised of soil erosion and runoff of Hg both naturally present and atmospherically deposited. Background releases, as defined here, do not represent the pre-industrial flux. We estimate these releases based on range of Hg loads (0.1–4 μg·m^−2^·a^−1^) reported in remote and pristine environments [31,32,33]. We scale up the estimate globally by multiplying various ranges of Hg loads by the size of individual drainage basin. We normalize Hg loads based on basin specific average total suspended solid (TSS) loads obtained from Ludwig et al. [34]. We use the natural breaks (Jenks) classification method to group drainage basins into four sediment yield classes (<30, 30–80, 80–130 and >130 Mg TSS·km^−2^) for which we apply the following Hg load ranges: 0.1–1, 1–2, 2–3 and 3–4 µg·Hg·m^−2^.

### 2.3. Remobilization from Terrestrial Systems

Our remobilization source category consists of Hg previously deposited to or accumulated in various environmental compartments as a result of natural processes augmented by anthropogenic activities. Within this source category we consider remobilization from contaminated surfaces via soil erosion and leaching and enhanced Hg releases and accumulation as a result of land and water management practices (agriculture and silviculture).

We define contaminated surfaces as sites with elevated Hg content surrounding locations where Hg was used or was/is present as a result of mining activities and industrial processes. We account for the following major sources: primary Hg mining, large-scale gold mining, non-ferrous metal processing (NFMP) of Pb, Zn and Cu, and various other polluted industrial sites where Hg is or was used as a catalyst (chlor-alkali, acetaldehyde, vinyl acetate and PVC plants). We distribute releases associated with mining activities along the locations of Hg mines obtained from mineral resources data system (MRDS) [35] and gold deposits [36], similar to the approach used by Selin et al. [37] for distribution of geogenic Hg emissions. A 10 km buffer zone surrounding Hg and Au mines was used to delineate the contributing area, based on reported extent of pollution in various studies [38,39,40,41]. Different ranges of Hg releases were then considered depending on the climatic position of the site: 10–100, 100–1000 and 1000–3000 g·Hg·km^−2^·a^−1^ in case of primary Hg mining [41,42] for arid, temperate and humid climate zone [30], respectively, while order of magnitude lower loads were considered for Au mining sites. For large Hg mining areas in China [43] and Slovenia missing in the MRDS the upper range of releases was used. For sites surrounding point sources (ore processing locations were adopted from USGS [35] and industrial installations intentionally using Hg as a catalyst either currently or in the past were compiled from various sources [11,44,45,46]), site-specific sediment yields were extracted from the GIS map of sediment flux [47] and combined with the extent of pollution as reported for various case studies. A uniform value of 3 km radius was used for selecting the size of the contributing area for each of these point sources, while soil Hg content in 0.1–0.3 µg·g^−1^ and 0.2–0.5 µg·g^−1^ range was selected for ore processing and sites with Hg used as catalysts, respectively [48,49,50]. For all of the contaminated systems considered here we assume the “background” contribution negligible.

We discuss potential Hg releases associated with land (agriculture and silviculture) management practices lacking detailed quantitative information based on the scarce information available and by using surrogate parameters (e.g., water consumption, hydro-meteorological conditions, land use).

## 3. Results and Discussion

### 3.1. Releases of Hg to Aquatic Systems

#### 3.1.1. Primary Releases of Anthropogenic Hg

**Figure 2:**
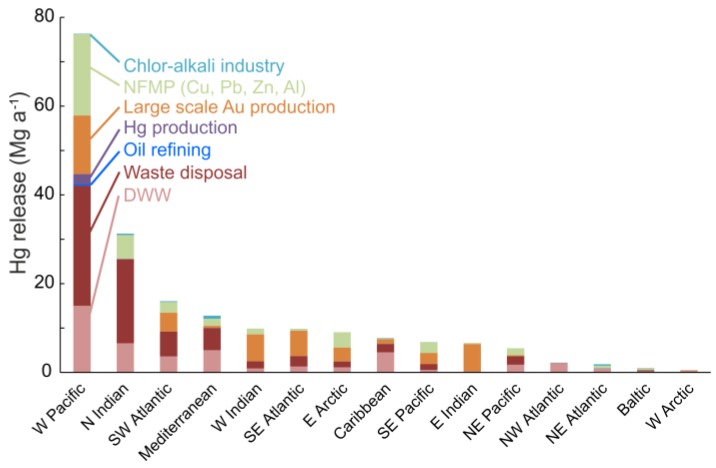
Release estimates of mercury (Hg) from various point source categories and by drainage basins in 2010 (NFMP: Non-ferrous metal production; DWW: Domestic wastewater).

In Figure 2, we show releases of Hg to aquatic systems by drainage basins and sector. Using the UNEP Toolkit approach, we estimate direct Hg discharges associated with industrial water effluents and disposal of Hg-containing products as 168 Mg·a^−1^ globally (range, 36–521 Mg·a^−1^). The majority (43%) is associated with Hg-containing products disposal, the rest being distributed among non-ferrous metal production facilities, including large scale gold mining, with only two percent of the releases from oil refining and chlor-alkali production. The majority (51%) of global Hg releases from these sources are from industrial installations and Hg-containing waste located in China and India, and draining to the West Pacific and North Indian Oceans (Figure 2). The wide range in the estimates reflects uncertainties related to those for atmospheric emission used to derive releases to water: activity data used, emission factors and assumptions made regarding applied technologies, as discussed in detail in AMAP/UNEP [18]. 

In addition to industrial sectors, domestic wastewater (DWW) is another important point source of Hg. We estimate the global amount of Hg entering aquatic systems from this source at 48 Mg·a^−1^ (range, 16–81 Mg·a^−1^). The low-high range reflects the range of wastewater Hg concentrations used to derive the estimate. Technology presently exists for Hg removal during treatment with efficiencies exceeding 98% [26,27]. However, even if treated, substantial amounts of Hg can be released from wastewater treatment plants (WWTPs) to local water bodies due to the large volumes of water processed (e.g., [22,51,52]). Due to the lack of treatment and high water consumption, almost half (45%) of estimated global releases associated with domestic wastewater are from densely populated regions draining to the North Indian and West Pacific oceans (Figure 2). The magnitude of Hg discharged from WWTPs is related to the type and the amount of Hg containing products in the waste stream. The phase out of many Hg containing products under the Minamata Convention could thus substantially decrease WWTP-associated aquatic Hg releases in the future. Constantly improved wastewater treatment practices are also expected to result in gradual decrease from this source, as already seen in North America [52] and China [22].

The overall magnitude of our release estimates is comparable with those obtained using independent approaches and assumptions. Present day releases from commercial Hg to water estimated by Horowitz et al. [7] are comparable to those estimated here, despite some minor differences in the sectors included in each inventory. Our estimates for individual sectors are further comparable with industry reports and other release estimates. For example, the OSPAR Commission (the governing body for the Convention for the Protection of the Marine Environment of the North-East Atlantic) reported 0.14 Mg of Hg released with waste water from 29 chlor-alkali plants located within the OSPAR region in 2009 [53]. Applying this value to 39 chlor-alkali plants that were using Hg cell technologies in the 27 EU Member States in 2010 [46] would result in an annual release of 0.18 Mg from this sector. Similarly, the World Chlorine Council [54] reports 0.3–0.8 Mg·Hg·a^−1^ released to water globally in the 2002–2013 period. These estimates are comparable to the calculated 0.65 Mg·a^−1^ using the Toolkit approach. For the oil refining sector, a wide range of values for Hg releases to water are reported for different regions in various databases/registers: for example, 0.03–0.36 kg/facility in 2011 in Australia [55], 0.2–1.28 kg/facility in 2010 in Canada [56] and 645 kg from 17 mineral oil and gas refineries in Europe in 2010 [57]. Considering these ranges and applying a rather conservative value of 0.5 kg·a^−1^ of Hg released from over 650 oil refineries globally [58] would approximate to 0.3 Mg·a^−1^, which is comparable to the 0.6 Mg·a^−1^ using the Toolkit approach. 

**Figure 3:**
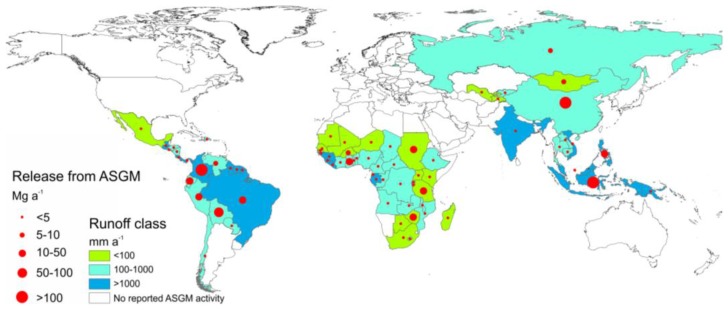
Estimated releases of Hg from artisanal and small-scale gold mining (ASGM) to terrestrial systems (land and water) and surface runoff class [30] for countries with known ASGM activities.

Artisanal and small-scale gold mining (ASGM), the largest source of anthropogenic Hg emissions [18], is another important source of Hg also for aquatic systems, with severe environmental impacts documented in many studies [29]. In Figure 3, we present the annual amount of Hg released to local rivers, lakes, soils and tailings each year for countries with reported ASGM activities. Information on the amounts released for an individual country is adopted from the most recent inventory [18], reporting average ASGM releases to terrestrial systems (land and water) in 2010 at 880 Mg·a^−1^ globally (range, 500–1260 Mg·a^−1^). Low-high range for this estimate is derived based on assumed Hg use and evaluation of the quality of the available information base for the various countries [18]. These numbers suggest that ASGM can be considered as the single largest anthropogenic source of Hg to aquatic systems on a global scale. This is due to both direct releases associated with its current use as well as to remobilization of Hg historically accumulated in areas where ASGM activities are/were conducted. 

The proportion of Hg directly released to local aquatic systems and the amount later remobilized from terrestrial ecosystems remains unknown. The latter depends on the hydrology of the mining region. Studies investigating environmental fate of Hg used in historical gold mining operations [59,60] suggest that a large proportion of the Hg has been remobilized over many years. The actual hydrologic Hg load away from ASGM operation sites might be dominated by the disturbance and mobilization of Hg rich sediment and floodplain soil during mining operations [61]. By classifying countries with ASGM activities according to climate zone, we find that many countries with the highest ASGM activity and Hg releases are located in regions susceptible to soil erosion (Figure 3). This includes Colombia, Indonesia, Philippines, Brazil, Guyana, Vietnam, Papua New Guinea, French Guiana, Suriname and Malaysia. Together these countries contribute more than 36% (~320 Mg·a^−1^) of global annual releases to terrestrial compartments from ASGM [18]. In some countries such as Democratic Republic of Congo, Uganda, Rwanda, Tanzania, Kenya, Burundi, and most other countries in the African Great Lakes Region, contaminated tailings are discharged directly into waterways. Following Horowitz et al. [7], for subsequent calculations and comparisons we assume 50% of total ASGM releases to terrestrial systems are released directly to water.

#### 3.1.2. Background Terrestrial Releases

We estimate global present-day river discharge of 170 to 300 Mg·Hg·a^−1^ as a result of erosion and runoff of Hg naturally present in soils or accumulated through atmospheric deposition. Low-high ranges reflect uncertainty in Hg loads used to derive this estimate. Using a mean all-time enrichment factor of two for terrestrial reservoirs [1], we estimate 50% of this is natural Hg. This natural contribution is even smaller in some compartments of the terrestrial environment such are labile organic soils [62]. Recent findings suggest an order of magnitude lower crustal abundance of Hg than previously estimated [63]. However, using uniform basin-scale Hg yields we do not capture elevated inputs in areas more prone to erosion or areas naturally enriched in Hg such as Hg mineral belts and tectonically active areas. These local/regional inputs are not expected to have a substantial affect globally. Our global estimate is lower than previous estimates that used different assumptions [10,18] but similar to Mason [64], who used crustal material and river water relationships between Hg and other elements (Na, Si) that have not been strongly impacted by anthropogenic activities to estimate the prehistorical Hg river flux at 260 Mg·a^−1^. The AMAP/UNEP [18] study based their estimate (320–960 Mg·a^−1^) on a range of background Hg concentrations in sediment (40 ± 20 ng·g^−1^) combined with modeled sediment yield [34]. Amos et al. [10] estimated background releases (760 Mg·a^−1^) using Hg measurements from Arctic rivers. While there is still considerable uncertainty in the estimate of “background” terrestrial release, the independent estimates converge to suggest that this “background” contribution is small relative to total present-day discharges of Hg from rivers (2000–5500 Mg·a^−1^) [9,10].

#### 3.1.3. Remobilization of Hg from Contaminated Terrestrial Systems

We estimate the contribution of Hg to nearby aquatic systems from all contaminated sites at 44 Mg·a^−1^ (range, 13–76 Mg·a^−1^). The estimated range is based on the reported range of Hg loads from areas where mining activities were conducted, and the magnitude and the spatial extent of contamination surrounding industrial installations. Using this approach, the vast majority (97%) of present-day releases is attributed to present and past Hg and Au mining, and only a minor share originates from ore processing and other industrial sites. Contamination associated with current industrial use of Hg is increasingly prevalent in Asia [18], while the majority of mining related releases are associated with historic Hg and precious metals (Au and Ag) mining areas in both Americas. Our Hg loads are adjusted to be representative for site-specific conditions using climate zone [30] and modeled sediment yields [47], respectively. Consequently, the magnitude of calculated loads depends mainly on local hydro-meteorological conditions and can vary from site to site by several orders of magnitude. For example, estimated annual Hg releases vary from 5 g to over 10 kg for NFMP sites and from less than 10 g to over 40 kg for locations using Hg as a catalyst, respectively. This is supported by observations in contaminated catchments [41,42,65]. At all these sites Hg is dispersed over large areas for which exact spatial distribution is unknown. The choice of the extent of contamination used in our estimates is 10 km radius for mining and 3 km for other sites. A ± 50% change in selected contamination extent would extend the bounds of the estimate to be between 3 and 150 Mg·a^−1^. While some of the contaminated systems discussed here might already be remediated, there might be other sources and activities resulting in potential contamination of surrounding environments. These are not considered here because of a lack of substantive information about their distribution and magnitude. 

#### 3.1.4. Releases Associated with Land Management Practices 

Land management practices can be responsible for enhanced mobility and release of Hg to water from various diffuse sources. For example, practices used in forestry are known to cause increased erosion and changed hydrological pathways and yield through the catchment soils, resulting in increased transport of Hg [66,67,68,69]. These processes are even more pronounced when forest is converted to agricultural land use, exposing the mineral soil horizon thus enhancing and accelerating Hg leaching [70]. Soil Hg loss occurs rapidly after deforestation [71], and data suggest that subsequent loss of the organic soil horizon is expected to mobilize 200–4600 μg·Hg·m^−2^ to Amazonian rivers [72]. Accelerated erosion caused by deforestation may cause both a sudden elevated episodic release of Hg associated with loss of organic soils, and enhanced erosion for many years [73]. These processes are important in the Amazonian ecosystems where high levels of Hg are accumulated in soils [38]. Studies have estimated these soils may release up to 3000 µg Hg m^−2^ per cm of soil eroded [72]. Consistent with this assumption, Lacerda et al. [74] concluded that the remobilization of Hg naturally present in forest soils during conversion of forest to other land uses, and not historic ASGM mining, was responsible for relatively high Hg levels in these rivers. Globally, deforestation is enhanced across the pan-tropical regions, where 9.5 million km^2^ of tropical forest was converted to agricultural land [75]. If we adopt mobilization of 2000 μg·Hg·m^−2^ due to the loss of the organic horizon as an average value globally [72] and considering the reported area deforested globally (13 million ha·a^−1^ in the 2000s) [76], this would yield in 260 Mg·Hg·a^−1^ entering local freshwaters. However, systematic catchment-scale studies in these areas are needed for reliable global quantification of associated Hg releases. 

Quantitatively assessing Hg releases from agricultural activities globally is not possible due to data limitations. Intensively cultivated river basins are known to export more Hg than basins where other land uses dominate (see [77,78]). Historically, Hg has been widely used in world agriculture with application rates as high as 10–10000 g·Hg·ha^−1^ [79]. Although the use of mercurial compounds is now banned in most countries, some use was reported to have occurred in some parts of the world even over the past decade [80]. Use of sewage sludge as agricultural fertilizer can also spread large amounts of Hg on agricultural lands [81]. For example, such applications were estimated to release 4.4 Mg·Hg·a^−1^ in the European Union [82]. Mercury can be leached from sewage sludge even more than a decade after its application [83]. Agricultural activities might be of particular concern because in addition to natural runoff, leaching and transport of Hg to aquatic ecosystems is enhanced due to large amounts of water used for irrigation purposes. Globally over 2500 km^3^·a^−1^ of water is used for irrigation at over 200 × 10^6^ ha with most of the irrigation areas located in India, China and in the United States [84].

### 3.2. Freshwater Releases in the Context of Global Hg Cycle

In Table 1, we summarize global aquatic Hg release estimates for all the sources discussed above. Global anthropogenic Hg discharges from primary anthropogenic sources and contaminated systems sums to ~300–1300 Mg·Hg·a^−1^ (assuming 50% of total ASGM releases are released directly to water). This suggests anthropogenic sources are at least comparable, if not several fold higher, to the global flux of Hg into freshwater ecosystems from atmospheric deposition (300–600 Mg·a^−1^). The latter is estimated from global atmospheric deposition to terrestrial surfaces (3000 Mg·a^−1^) [17] and by assuming 10%–20% of total terrestrial deposition is typically released from catchments into rivers [31,77,85].

**Figure 4:**
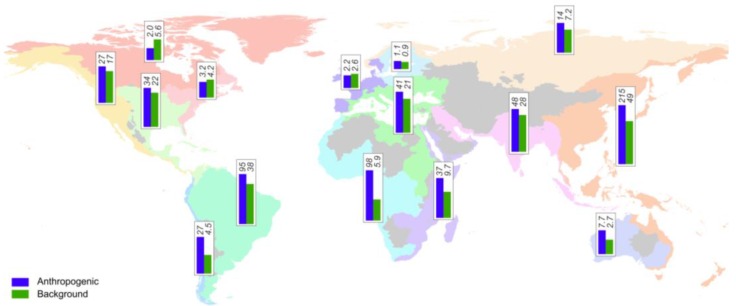
Sum of release estimates associated with anthropogenic activities (point sources, remobilization from contaminated systems and artisanal and small-scale gold mining ASGM) and background Hg release estimates for individual drainage basin. In the case of the sum of anthropogenic sources, we consider proportion of Hg released to land versus water in ASGM operations to be 50%. All units are in Mg·a^−1^. For illustrative purpose we use log(x + 1) transformation to draw the bars.

Compared to atmospheric Hg inputs, terrestrial sources discussed here are unevenly distributed within catchments and can have more significant local impact. On a basin-wide scale, there are substantial differences in magnitude and relative importance of individual sources. In Figure 4 and Table S3, we compare average releases associated with background Hg and anthropogenic activities (point sources, remobilization from contaminated systems and ASGM). Background Hg releases are highest in areas where natural conditions favor soil erosion and sediment delivery. The most important transporters are rivers draining Southeast Asia and high-standing islands of Oceania, which also deliver 70% of the sediment discharge to the global ocean [86]. The most significant present-day releases relative to background releases are in areas with the largest number of both historic and present-day anthropogenic sources draining South and Southeast Asia and both Americas. Some of these areas coincide with areas of high freshwater and sediment discharge. This results in the predominant discharge of Hg from these regions to oceans globally, as established in previous studies [8,9,10].

Once released to an aquatic system, the downstream fate of Hg remains largely unknown. Important processes include sedimentation and remobilization of Hg previously deposited within river systems. Retention behind various artificial impoundments can also play a significant role in preventing Hg associated with river sediments from reaching coastal waters. For example, according to Milliman and Farnsworth [86], the estimated reduction in sediment load due to reservoir construction in the Mediterranean region is ~80%, and to a similar extent in Asia (~70% reduction for China’s major rivers), and as Hg is mostly transported as particulate these changes will have markedly impacted Hg dynamics in coastal and shelf regions [87]. Due to substantial Hg transport by Asian rivers [10,16], the majority of Hg retention by impoundments is expected to occur in basins draining to northern Indian and western Pacific seas.

Accumulation of Hg carried by rivers can also be important in endorheic regions, which cover 10% of the surface of continents [88]. A known example is Lake Ontario within the Laurentian Great Lakes, where Hg contamination is a result of historical loadings from industrial activities in the drainage area in combination with enhanced atmospheric deposition [89,90]. Our estimates suggest ~20 Mg·a^−1^ of Hg from anthropogenic point sources (ASGM not included) is released to such closed basins globally. On the other hand, some industries located near the coast and discharging Hg directly to coastal waters can also be important locally (e.g., [91,92]). 

We further compare our estimated Hg releases to freshwaters with the amount of Hg reaching coastal margins. Summing all Hg discharges discussed in this work (Table 1; total ~500–1600 Mg·a^−1^, assuming 50% of total ASGM releases are released directly to water) with Hg supplied as a result of runoff from atmospheric deposition (300–600 Mg·Hg·a^−1^), results in total of ~800–2200 Mg·Hg·a^−1^ entering freshwater systems. This input is lower than the amount of Hg entering global coastal margins estimated from a review of measured concentrations in river waters by Amos et al. [10] (5500 ± 2700 Mg·a^−1^). This difference cannot be explained by submarine groundwater discharge (SGD) only, which were estimated previously to be <800 Mg·a^−1^ [93]. Earlier estimates of inputs to coastal margins are more comparable (1000–2700 Mg·a^−1^) [8,9]. We postulate that differences among estimates reflect temporal lags in Hg releases through watersheds and that much of load presently discharged to ocean margins is legacy Hg. Although the estimates are not directly comparable, a large gap between the estimates outside the full uncertainty range of each indicates (i) the need for future research to identify the potential missing sources of Hg to rivers currently not quantified/accounted for in this analysis (e.g., urban runoff [94], coal mining and washing, water use in coal-fired power plants [22], various land management activities etc.), (ii) the need to further account for the complexity of Hg distribution within both terrestrial and freshwater systems (including deposition/remobilization patterns) all the way from its source to its ultimate discharge to coastal margins, and (iii) to additional monitoring data with better spatial coverage on present-day Hg discharges from rivers to ocean margins.

## 4. Conclusions

We present here an important first step towards the assessment of global Hg releases to freshwater aquatic environments. Quantitative inventories are a prerequisite for discussion of the fate and transport of Hg and amounts converted into the bioaccumulative species, methylmercury. Although freshwaters addressed here represent only a minor share of the global water budget, the contributions of Hg to these environments is of great importance because of their relevance for human and ecological exposures. This knowledge is important for investigation of ecosystem responses to changes in Hg loads [95], particularly in ecosystems susceptible to Hg methylation and bioaccumulation [96,97,98]. However, insufficient data and unquantifiable sources drive large uncertainty in the estimates in our inventory. In the case of current primary anthropogenic Hg sources, the UNEP Toolkit approach used here has the potential to underestimate the releases to water. These unaccounted-for releases might be associated with activities where air emissions are insignificant and thus there are no corresponding emissions included in the air emissions inventory. Moreover, there are significant uncertainties related to the validity and utility of the Toolkit distribution factors. In the case of remobilization of Hg from contaminated systems, releases from this category might be underestimated, as there are numerous areas globally where Hg is/was used in various products and processes (e.g., pulp and paper mills, paint, lamps, batteries and explosive production, etc.) and for which locations and the extent of contamination are unknown. Similarly, important sectors like Hg releases associated with land management practices and other diffuse sources are presently understudied. In order to improve the estimates presented here, a systematic and harmonized monitoring as well as reporting of Hg releases is needed. This is especially critical for diffuse releases associated with legacy Hg, as these releases are much harder to predict. Therefore, we suggest expanding current observing systems that are global in nature (e.g., Global Mercury Observation System [99]) to focus on freshwater inputs in addition to their current focus on atmospheric sources.

## Figures and Tables

**Table 1:** Summary of release estimates from various Hg sources to freshwater aquatic environments globally.

Source	Average (Range) Mg·a^−1^
Background terrestrial	230 (170–300)
Primary anthropogenic	
*- Point sources*	220 (50–600)
- ASGM *	880 (500–1260)
Remobilization from contaminated systems	40 (10–80)

* To both land and water; for comparisons with other inputs we assume 50% is released directly to water.

## Supplementary Materials

The following are available online at www.mdpi.com/1660-4601/14/2/138/S1. Figure S1: Schematic approach for calculation and distribution of aquatic Hg release estimates from the atmospheric Hg emission inventory, Table S1: Values used to derive aquatic Hg release estimates from the atmospheric Hg emission inventory, Table S2: Summary of data used to derive estimates discussed in the manuscript, Table S3: Release estimates (Mg·a^−1^) from various Hg sources to individual drainage basin.
