# Supplementary material for: Toward an Assessment of the Global Inventory of Present-Day Mercury Releases to Freshwater Environments
Source: Int J Environ Res Public Health. 2017 Feb 1;14(2):138. doi: 10.3390/ijerph14020138 (PMC5334692; doi:10.3390/ijerph14020138)
Supplement: Supplementary file 1 [file ijerph-14-00138-s001.pdf]

Supplementary Materials: Toward an Assessment of the Global Inventory of Present-Day Mercury Releases to Freshwater Environments

David Kocman, Simon J. Wilson, Helen M. Amos, Kevin H. Telmer, Frits Steenhuisen, Elsie M. Sunderland, Robert P. Mason, Peter Outridge and Milena Horvat

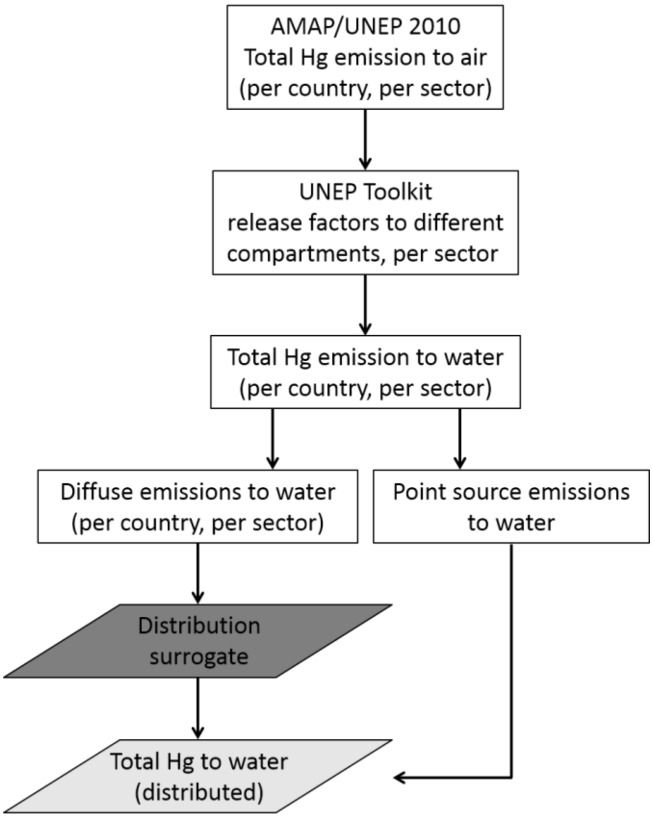

Figure S1. Schematic approach for calculation and distribution of aquatic Hg release estimates from the atmospheric Hg emission inventory.

Table S1. Values used to derive aquatic Hg release estimates from the atmospheric Hg emission inventory.

Sector	Estimated Hg Emission to air ($Mg \cdot a^{-1}$) [1]	UNEP Toolkit Distribution Factor [2]		Scaling Factor (Water/Air)	Estimated Hg Release to Water ($Mg \cdot a^{-1}$)
		to air	to water		
Chlor-alkali industry	28	0.1	0.01	0.1	2.8
Oil refining	16	0.25	0.01	0.04	0.6
Large scale Au	97	0.04	0.02	0.5	49
Non-ferrous metal production (Cu, Pb, Zn)	189	0.1	0.02	0.2	38
Non-ferrous metal production (Al)	4.9	0.15	0.1	0.67	3.3
Non-ferrous metal production (Hg)	11	0.25	0.06	0.24	2.6
Disposal of Hg-containing products	89				72
—uncontrolled landfill		0.1	0.1	1	
—controlled landfill		0.1	0.001	0.01	
Sum					168 *

* In addition to sectors covered by UNEP Toolkit, releases associated with domestic wastewater (DWW) are estimated at 48 Mg a^{-1}.

Table S2. Summary of data used to derive estimates discussed in the manuscript.

Category/Source	Range of Hg Concentration/Load	Ancillary Data
Domestic wastewater	Untreated wastewater: 100-500 ng·Hg·L^{-1}, Treated wastewater 5–20 ng·Hg·L^{-1} [3–5]	Wastewater volumes [6] Treatment practices [7,8]
Artisanal and small-scale gold mining	Annual amount of Hg released to land and water in individual country (500-1260 Mg·Hg·a^{-1} globally) [1]	Climatic zone of a country [9]
Background releases	0.1–4 μg Hg m^{-2}·a^{-1} [10–12]	Riverine sediment fluxes [13]
Remobilization from terrestrial systems	Primary Hg mining: 10–3000 μg Hg m^{-2}·a^{-1} [14,15] Large-scale Au mining: 1–300 μg Hg m^{-2}·a^{-1} Industrial installations [16–18]: 0.1–0.3 μg Hg·g^{-1} in soil (NFMP sites) 0.2-0.5 μg Hg·g^{-1} in soil (Hg catalyst)	Climatic zone of mining area [9] Global Hg mining areas [19,20] Global gold deposits [21] Locations of industrial installations [19,22–25] Site-specific sediment yield [26]

Table S3. Release estimates (Mg·a^{-1}) from various Hg sources to individual drainage basin.

Basin/Source Category	Background	Point Source	ASGM *	Remobilization from contaminated Systems
Baltic	0.92	1.01	0.02	0.07
Caribbean	21.6	7.72	47.8	2.49
E Arctic	7.16	9.05	8.19	0.82
E Indian	2.73	6.64	1.37	0.42
Endoreic	14.1	19.3	54.1	3.56
Mediterranean	21.1	12.8	52.0	2.34
N Indian	27.6	31.3	32.3	0.96
NE Atlantic	2.65	1.85	0.00	0.33
NE Pacific	17.0	5.46	6.25	18.3
NW Atlantic	4.17	2.20	0.00	0.98
SE Atlantic	5.87	9.82	175	0.38
SE Pacific	4.50	6.87	39.0	0.77
SW Atlantic	37.9	16.1	154	1.46
W Arctic	5.60	0.52	0.00	1.45
W Indian	9.69	9.87	52.6	0.48
W Pacific	49.5	76.3	258	9.70
SUM	232	217	881	44.5

* To both land and water.

References

1. AMAP/UNEP. *Technical Background Report for the Global Mercury Assessment 2013*; Arctic Monitoring and Assessment Program: Oslo, Norway; UNEP Chemicals Branch: Geneva, Switzerland, 2013.
2. UNEP. *Toolkit for Identification and Quantification of Mercury Sources, Guideline for Inventory Level 1*, version 1.2; UNEP Chemicals Branch: Geneva, Switzerland, 2013.
3. Mugan, T.J. Quantification of total mercury discharges from publicly owned treatment works to Wisconsin surface waters. *Water Environ. Res.* **1996**, *68*, 229–234.
4. Balogh, S.J.; Nollet, Y.H. Mercury mass balance at a wastewater treatment plant employing sludge incineration with offgas mercury control. *Sci. Total Environ.* **2008**, *389*, 125–131.
5. Dean, J.D.; Mason, R. *Estimation of Mercury Bioaccumulation Potential from Wastewater Treatment Plants in Receiving Waters: Phase 1*; WERF Report 05-WEM-1CO; IWA Publishing: London, UK, 2009.

6. GWSP. Digital Water Atlas, Map 46: Domestic Water Use (V1.0). Available online: http://atlas.gwsp.org (accessed on 30 January 2017).
7. UNEP. *The State of the Marine Environment—Trends and Processes;* United Nations Environment Programme and the Global Programme of Action for the Protection of the Marine Environment from Land-based Activities (GPA) of the United Nations Environment Programme (UNEP); UNEP/GPA Coordination Office: The Hague, Netherlands, 2006.
8. Sato, T.; Qadir, M.; Yamamoto, S.; Endo, T.; Zahoor, A. Global, regional, and country level need for data on wastewater generation, treatment, and use. *Agric. Water Manag*. **2013**, *130*, 1–13.
9. Fekete, B.M.; Vörösmarty, C.J.; Grabs, W. *Global Composite Runoff Fields Based on Observed River Discharge and Simulated Water Balances*; UNH/GRDC Composite Runoff Fields v1.0; Complex Systems Research Center, University of New Hampshire: Durham, NH, USA, 2000.
10. Grigal, D.F. Inputs and outputs of mercury from terrestrial watersheds: A review. *Environ. Rev.* **2002**, *10*, 1–39.
11. Brigham, M.E.; Wentz, D.A; Aiken, G.R.; Krabbenhoft, D.P. Mercury Cycling in Stream Ecosystems. 1. Water Column Chemistry and Transport. *Environ. Sci. Technol.* **2009**, *43*, 2720–2725.
12. Schuster, P.F.; Striegl, R.G.; Aiken, G.R.; Krabbenhoft, D.P.; Dewild, J.F.; Butler, K.; Kamark, B.; Dornblaser, M. Mercury Export from the Yukon River Basin and Potential Response to a Changing Climate. *Environ. Sci. Technol.* **2011**, *45*, 9262–9267.
13. Ludwig, W.; Amiotte-Suchet, P.; Probst, J.L. ISLSCP Initiative II Collection. Data Set. In *ISLSCP II Global River Fluxes of Carbon and Sediments to the Ocean*; Hall, F.G., Collatz, G., Meeson, B., Los, S., Brown de Colstoun, E., Landis, D., Eds.; Oak Ridge National Laboratory Distributed Active Archive Center: Oak Ridge, TN, USA, 2011.
14. Dai, Z.H.; Feng, X.B.; Zhang, C.; Shang, L.H.; Qiu, G.L. Assessment of mercury erosion by surface water in Wanshan mercury mining area. *Environ. Res.* **2013**, *125*, 2–11.
15. Tetra Tech. *Guadalupe River Watershed Mercury TMDL Project*; Final Conceptual Model; Prepared for San Francisco Bay Regional Water Quality Control Board: Lafayette, CA, USA, 2005.
16. Biester, H.; Muller, G.; Scholer, H.F. Estimating distribution and retention of mercury in three different soils contaminated by emissions from chlor-alkali plants: Part I. *Sci. Total Environ.* **2002**, *284*, 177–189.
17. Sterckeman, T.; Douay, F.; Proix, N.; Fourrier, H.; Perdrix, E. Assessment of the contamination of cultivated soils by eighteen trace elements around smelters in the North of France. *Water Air Soil Pollut.* **2002**, *135*, 173–194.
18. Li, G.H.; Feng, X.B.; Qiu, G.L.; Bi, X.Y.; Li, Z.G.; Zhang, C.; Wang, D.Y.; Shang, L.H.; Guo, Y.N. Environmental mercury contamination of an artisanal zinc smelting area in Weining County, Guizhou, China. *Environ. Pollut.* **2008**, *154*, 21–31.
19. USGS. *Mineral Resources Data System*; US Geological Survey: Reston, VA, USA, 2005. Available online: http://mrdata.usgs.gov/mrds (accessed on 30 January 2017).
20. Feng, X.; Qiu, G. Mercury pollution in Guizhou, Southwestern China—An overview. *Sci. Total Environ.* **2008**, *400*, 227–237.
21. Gosselin, P; Dube, B. *Gold Deposits of the World: Distribution, Geological Parameters and Gold Content*; Open File 4895; Geological Survey of Canada: Quebec City, QC, Canada, 2005.
22. Kocman, D.; Horvat, M.; Pirrone, N.; Cinnirella, S. Contribution of contaminated sites to the global mercury budget. *Environ. Res.* **2013**, *125*, 160–170.
23. Trip, L.; Thorleifson, M. *The Canadian Mercury Cell Chlor-alkali Industry: Mercury Emissions and Status of Facilities 1935–1996*; Report to Transboundary Air Issues Branch; Environment Canada: Hull, QC, Canada, 1998.
24. Mahan, S.; Savitz, J. *Cleaning up: Taking Mercury-Free Chlorine Production to the Bank*; Oceana: Washington, DC, USA, 2007.
25. UNEP. Global Estimate of Global Mercury Cell Chlorine Capacity for 2010. Available online: http://web.unep.org/chemicalsandwaste/global-mercury-partnership/mercury-reduction-chlor-alkali-sector/reports-and-publications (accessed on 30 January 2017).

26. Ludwig, W.; Amiotte-Suchet, P.; Probst, J.L. ISLSCP Initiative II Collection. Data set. In *ISLSCP II Atmospheric Carbon Dioxide Consumption by Continental Erosion*; Hall, F.G., Collatz, G., Meeson, B., Los, S., Brown de Colstoun, E., Landis, D., Eds.; Oak Ridge National Laboratory Distributed Active Archive Center: Oak Ridge, TN, USA, 2011.

© 2017 by the authors; licensee MDPI, Basel, Switzerland. This article is an open access article distributed under the terms and conditions of the Creative Commons Attribution (CC-BY) license (http://creativecommons.org/licenses/by/4.0/).
